# Exploring the role and mechanisms of the *PMA* gene in *Aspergillus fumigatus*

**DOI:** 10.1080/21501203.2024.2354273

**Published:** 2024-06-03

**Authors:** Chengrui Tan, Shaojie Jiang, Hongli Zhai, Qingwen Hu, Chenxi Liu, Yi Sun, Lujuan Gao

**Affiliations:** a Department of Gastroenterology, Jingzhou Hospital Affiliated to Yangtze University, Jingzhou, China; b Health Science Center, Yangtze University, Jingzhou, China; c Department of Dermatology, Jingzhou Hospital Affiliated to Yangtze University, Jingzhou, China; d Department of Dermatology, Zhongshan Hospital (Xiamen), Fudan University, Xiamen, China; e Department of Dermatology, Zhongshan Hospital, Fudan University, Shanghai, China; f Xiamen Clinical Research Center for Cancer Therapy, Xiamen, China

**Keywords:** *Aspergillus fumigatus*, invasive aspergillosis, plasma membrane proton ATPase, antifungal drug resistance, voriconazole, PMA knockout mutant

## Abstract

In the realm of aspergillosis, a critical concern for immunocompromised patients facing *Aspergillus fumigatus*, effective management hinges on understanding fungal growth, stress resistance, and response to antifungal treatments. Our study investigates the crucial role of fungal plasma membrane proton ATPase (PMA) in nutrient absorption, intertwined with growth and antifungal susceptibility. We employed a high-throughput knockout method to create the PMA gene knockout mutant, Δ*Afu-PMA1*, in *A. fumigatus*, alongside a complementation strain. Antifungal susceptibility to triazoles was assessed by micro-dilution method and E-test, revealing decreased sensitivity to voriconazole in Δ*Afu-PMA1*. Comparative analysis demonstrated significant growth differences, with wild-type strain surpassing Δ*Afu-PMA1* by 3.2-fold. Under oxidative stress and heightened osmotic pressure, Δ*Afu-PMA1* showed notable growth defects. Loss of PMA led to increased ergosterol and decreased ATP content, alongside pH changes in the culture medium. Transcriptome sequencing unveiled revealed a reduced expression of genes associated with ribosome function, the MAPK pathway, endoplasmic reticulum, and the transport and metabolism of fats, sugars, and proteins in Δ*Afu-PMA1*, highlighting PMA’s regulatory role in growth and adaptation. These findings emphasise PMA as a potential target for future antifungal drugs, offering hope in combating aspergillosis.

## 1. Introduction

*Aspergillus fumigatus* is a prominent fungal species responsible for invasive fungal infections (IFIs). Invading fungal infections frequently occur in individuals with compromised or weakened immune systems due to long-term chemotherapy, prolonged use of glucocorticoids, and viral infections. It is estimated that around 2 million people worldwide contract IFIs each year, with mortality rates soaring as high as 95% (Clausen et al. 2017; Kjellerup et al. 2017). The rising incidence of fungal infections, coupled with the improper utilisation of antifungal medications, has led to a growing prevalence of fungal resistance. Notably, resistance to triazole drugs in *A. fumigatus* has emerged as a significant clinical concern in multiple regions, including Europe, China, Canada, and the United States (Pfaller et al. 2024). The drug resistance is associated with an increased expression of *CYP51A*, catalase (Cat1), enolase, thioredoxin peroxide, and a decreased expression of cytochrome C, RodA, and PhiA-related proteins, as well as mutations of *CYP51A* (Shishodia et al. 2019). Over the past three years, there has been an increase in the occurrence of *A. fumigatus* infections, mainly linked to the widespread of the novel coronavirus and the use of immunomodulatory medications throughout treatment (Koehler et al. 2022; Heung et al. 2023). Nevertheless, there has been a constrained advancement in the development of antifungal medications, and the three primary classes of drugs employed in clinical practice remain azoles, echinocandins, and polyenes. Plasma membrane H (+) ATPase (*PMA*) is a P-type ATPase encoded by the *PMA* gene, which can break down ATP and form an electrochemical gradient. Other transporters rely on this electrochemical gradient generated by plasma membrane H (+) ATPase to absorb ions and nutrients (Clausen et al. 2017; Kjellerup et al. 2017). Biochemical characterisation studies conducted on *Schizosaccharomyces pombe*, *Saccharomyces cerevisiae*, and *Neurospora crassa* have demonstrated that plasma membrane H (+) ATPase belongs to the class of P-type ATPases. This ATPase plays a critical role in preserving the dynamic pH equilibrium within the cell, overseeing the absorption of ions and nutrients, and participating in fungal growth and pathogenicity (Zhgun et al. 2020). Due to the high conservation of the *PMA* gene in fungi and the successful use of cardiac glycosides targeting the Na^+^K^+^-ATPase, as well as proton pump inhibitors targeting the gastric H^+^K^+^-ATPase in clinical applications, we anticipate that studying the *PMA* gene in *A. fumigatus* could provide insights into its growth resistance mechanisms (Kjellerup et al. 2017). This, in turn, could potentially present a novel approach to addressing antifungal resistance in clinical settings (Burghoorn et al. 2002). In this research, we conducted a comprehensive functional analysis of the *A. fumigatus PMA* gene, exploring its impact on growth, susceptibility to antifungal agents, and resistance to stress. We delved into the molecular mechanisms underlying these effects.

## 2. Materials and methods

### 2.1. Strains, plasmids, and culture conditions

The *A. fumigatus* strain A1160 (ΔKU80, *pyrG*-, obtained from the Fungal Genetics Stock Center) exhibits uracil deficiency. This particular strain served as the host for our experiments and was cultivated on Czapek Dox Medium supplemented with uracil (CZA+U) at 37 °C for 3–5 d. In our study, the control was represented by the wild-type strain (WT), which had undergone A1160 transformation with exogenous *pyr*G and was cultured under identical conditions. The plasmid pLAX223, containing the selection marker *pyr*G, was cultured on Luria-Bertani agar supplemented with ampicillin (1 μg/mL) at 37 °C with agitation at 120 r/min for a duration of 16 h (Ma et al. 2009).

### 2.2. Construction of the gene knockout cassette box

The gene was extracted from WT using the ® Fungal DNA Kit (OMEGAD3390-02) to amplify the target gene *PMA*, which has a size of approximately 1,200 bp. The upstream fragment was amplified using primers P1 and P2, while the downstream fragment was amplified using primers P3 and P4. Plasmid extraction was performed using the Plasmid Extraction Kit (19001ES50), and the pyrG-n-F and pyrG-n-R primers were used to amplify the selection marker fragment. To combine the upstream and downstream fragments with the selection marker fragment, we employed the nested primers *PMA* P5 and *PMA* P6. The details of the primer sequences are provided in Table 1. The PCR fusion system and amplification conditions for gene knockout were referenced from a previously described method (Zhao et al. 2019).

**Table 1.** Primer sequences used in this study.

Name	Sequence (5’-3’)	Function
PMA P1	CTGCTAAGGTGGATGTGGTTGTA	Amplify the left wing
PMA P2	TAGTTCTGTTACCGAGCCGGCCGACGAGGAGCGAAACACCACCT	
PMA P3	GCTCTGAACGATATGCTCCAACTATGCAACAAGCTAATGGAGTAG	Amplify the right wing
PMA P4	GTCCCTGCAACCGACGTTCCTGAG	
PMA P5	CTAACCGTATAGTACTAATGATA	Fusion PCR
PMA P6	AAGATGTAAGGATAATCGCTGAT	
pyrG-n-F	CCGGCTCGGTAACAGAACTACCGCAGACAATGCTCTCTATC	Amplify selection markers pyrG
pyrG-n-R	GTTGGAGCATATCGTTCAGAGCAATACCGTTACACATTTCCA	
Carslan F1	AGGATAACAAATAGCTGATGCGTA	Verify the positive transformation
Carslan R1	TACGCATCAGCTATTTGTTATCCT	
AIM-F	TTAACGTCGACCTCGAAGTTAGAGTATTCGGTGGTATG	Amplify the left wing
AIM-R	TCTCGCCACGTTCGCCTAGCGAGTTATGAGTGAAAGTG	Amplify the right wing
Kasel-F2	CTCGTCCATGCCGTGAGTGA	Complementation strains to positive transformants
Kasel-R2	GCCACAAGTTCAGCGTGTCC	

The underlined letters represent the linking fragments in the PCR fusion.

### 2.3. Preparation of protoplasts and transformation

The prepared fused fragments were introduced into A1160 using the protoplast transformation method (Liu et al. 2022). As *pyrG* was used as the selection marker, the successful transformation was determined by the presence of fungal growth on Czapek Dox Medium (CZA) in the experimental group and the absence of fungal growth in the negative control group. The successful knockout of the *PMA* gene resulted in the naming of the knockout strain as Δ*Afu-PMA1*.

### 2.4. Identification of the transduction candidate

Single colonies grown on CZA were picked with a pipette tip and transferred to another CZA medium. After these colonies had grown, they were subsequently transferred to a fresh CZA medium and allowed to culture for 3–4 d. The final colony that was actively growing was selected for DNA extraction, which was carried out using a DNA extraction kit. Subsequently, PCR amplification was conducted using the validation primers pyrG-v-R and pyrG-v-F, and the resulting band position was visualised using gel electrophoresis (1,500 bp). The remaining PCR products were sent for sequencing [Sangon Biotech (Shanghai) Co., Ltd., China].

### 2.5. Construction of the complementation strain

To construct the complementation strain, we performed gene amplification of the target gene and employed the method of protoplast transformation (Tan et al. 2023). Here is a summary: The flanking region and coding region of the *Afu-PMA* gene were amplified from AF293 genomic DNA using primers AIM-F and AIM-R. Unnecessary fragments were removed by restriction enzyme digestion, followed by cloning using the Hieff ClonePlus ONE Step Cloning Kit [Yeasen Biotechnology (Shanghai) Co., Ltd., China]. The recombinant plasmid product was then introduced using the protoplast transformation method. DNA from the resulting colonies was extracted and subjected to PCR using primers Kasel-F2 and Kasel-R2, and the resulting bands were visualised using a gel electrophoresis apparatus (500 bp). The primer sequences are provided in Table 1.

### 2.6. Phenotypic growth experiment

#### 2.6.1. Growth speed difference experiment

Δ*Afu-PMA1*, WT, and Δ*Afu-PMA1::Afu-PMA1+* strains grown on CZA medium for 3–5 d were transferred to sterile 1× phosphate buffered saline solution (PBS) using a sterile cotton swab and adjusted to a concentration of 1 × 10^6^ CFU/mL. Five microlitres of spore suspension were pipetted and spotted onto the CZA medium. After allowing the plates to dry for 15 min, they were incubated at 37 °C. The diameter of the *A. fumigatus* colony was measured at 3 time points, namely, 24 h, 48 h, and 72 h.

#### 2.6.2. Growth morphology difference experiment

Spore suspensions of Δ*Afu-PMA1*, WT, and Δ*Afu-PMA1::Afu-PMA1+* strains were prepared and adjusted to a concentration of 2 × 10^4^ CFU/mL. Cotton swabs were used to evenly spread the suspension on the Sabouraud dextrose agar (SDA). Following a 48-h incubation at 37 °C, colonies from the three strains were selected and transferred onto glass slides. A fungal fluorescence staining solution was added, and the morphology differences of *A. fumigatus* Δ*Afu-PMA1*, WT, and Δ*Afu-PMA1::Afu-PMA1+* were observed under a fluorescence microscope at a 40-fold magnification.

### 2.7. Antifungal sensitivity assay

#### 2.7.1. Microdilution method

The experimental procedure followed the guidelines outlined in the Clinical and Laboratory Standards Institute (CLSI) protocol M38-A2 for testing the antifungal susceptibility of filamentous fungi (Institute 2008). *Candida parapsilosis* ATCC 22019 and *Aspergillus flavus* ATCC 204304 were used as the quality control strains. The WT, Δ*Afu-PMA1*, and Δ*Afu-PMA1::Afu-PMA1+* strains were prepared, yielding a final spore concentration of 2 × 10^4^ CFU/mL. The tested drugs, which included itraconazole, voriconazole, and posaconazole, from Selleck Chemicals (USA), were prepared in eight different concentration gradients, with the highest concentration being 8 μg/mL, 8 μg/mL, and 4 μg/mL, respectively. Following a 48-h incubation period at 35 °C, the growth of fungal colonies on the 96-well plate was examined to determine the drug susceptibility results, and the minimum inhibitory concentration (MIC) values were recorded.

#### 2.7.2. E-test method

The mature WT, Δ*Afu-PMA1*, and Δ*Afu-PMA1::Afu-PMA1+* strains were prepared as spore suspensions with a concentration of 1 × 10^6^ CFU/mL. This experiment was conducted following the method described by Koga-Ito et al. (2008). Antifungal susceptibility testing strips of itraconazole (liofilchem ICZ 92148), voriconazole (Merck, VCZ 532800), and posaconazole (liofilchem PCZ 92152) were utilised. The sealed plates were then incubated at 35 °C for 48 h.

### 2.8. Oxidative stress assay

The oxidative stress experiment followed the specified operating procedure (Ma et al. 2012). In this study, the concentrations of the oxidising agent H_2_O_2_ and menadione were 5 mmol/L and 25 mmol/L, respectively. Δ*Afu-PMA1*, WT, and Δ*Afu-PMA1::Afu-PMA1+* were set at three different concentration gradients of 1 × 10^4^ CFU/mL, 1 × 10^5^ CFU/mL, and 1 × 10^6^ CFU/mL, respectively.

### 2.9. Osmotic stress assay

In the osmotic sensitivity experiment, we followed the previously described experimental protocol (Ma et al. 2009). Briefly, the concentrations of NaCl and D-sorbitol were 1 mmol/L and 0.5 mmol/L, respectively. Δ*Afu-PMA1*, WT, and Δ*Afu-PMA1::Afu-PMA1+* were set at three different concentration gradients of 1 × 10^4^ CFU/mL, 1 × 10^5^ CFU/mL, and 1 × 10^6^ CFU/mL, respectively.

### 2.10. Determination of ergosterol

The matured Δ*Afu-PMA1*, WT, and Δ*Afu-PMA1::Afu-PMA1+* were scraped off and weighed after culturing on CZA medium. Then, the extraction of ergosterol was performed following the method described by Arthington-Skaggs et al. (1999, 2002). The extracted product was subjected to full spectrum scanning using Nanodrop-one (Thermo Fisher Scientific), and the scanning graph was recorded between 240 and 300 nm. Samples were read at 2 nm intervals between 230 and 300 nm. The formula for calculating ergosterol is as follows:

$$\mathrm{Brassionosteroid}\;\mathrm{content}\text{\textbackslash{}break}=\left\{\left[\left(A281.5÷290\right)\times F\right]÷\mathrm{wet}\text{\textbackslash{}break}\;\mathrm{weight}-\left[\left(A230÷518\right)\times F\right]÷\mathrm{wet}\;\mathrm{weight}\right\}\times 100\%$$

where F is the factor for dilution in ethanol and 290 and 518 are the E values (in percentages per centimetre) determined for crystalline ergosterol and 24(28) DHE, respectively.

### 2.11. Determination of intracellular ATP levels

Mature colonies of Δ*Afu-PMA1*, WT, and Δ*Afu-PMA1::Afu-PMA1*+ strains grown on the SDA medium were collected by scraping with a sterile cotton swab. Approximately, 0.1 g of each sample was weighed and transferred into individual 1.5 mL Eppendorf tubes sterilised under high pressure. ATP extraction and measurement were conducted using the ATP Content Assay Kit (Beijing Boxbio Science & Technology Co., Ltd., China), following the provided kit instructions. Finally, ATP levels were calculated based on the formula provided in the kit’s instructions.

### 2.12. Experiment to determine the influence of pH on the environment

The procedure for preparing Δ*Afu-PMA1*, WT, and Δ*Afu-PMA1::Afu-PMA1+* fungal suspension is the same as before. Add the prepared fungal suspensions separately into 10 mL of Sabouraud Dextrose Broth (SAB) to achieve a final concentration of 1 × 10^4^ CFU/mL. Set the incubator temperature to 37 °C with shaking at 120 r/min. Use a pH metre to measure the pH values of each sample at 0 h, 6 h, 12 h, and 24 h, and record them. Repeat this experiment on different days three times.

### 2.13. RNA sequencing

Total RNA was extracted following the guidelines provided for TRlzol Reagent (Life Technologies, California, USA). A sequencing library was generated using the Hieff NGS Ultima Dual-mode mRNA Library Prep Kit for Illumina [Yeasen Biotechnology (Shanghai) Co., Ltd., China]. Sequencing was carried out by Biomarker Technologies Co., Ltd in Beijing, China. Further analysis and annotation were conducted using AF293 as the reference genome, focusing exclusively on sequences that matched completely or with one mismatch, as per the reference genome. Gene function annotation was performed using the Clusters of Orthologous Groups of proteins (COG) and the Kyoto Encyclopedia of Genes and Genomes (KEGG) Ortholog database (KO) (Ju et al. 2024). The experiment was repeated 3 times.

### 2.14. Data processing

GraphPad Prism 8 was used for drawing graphs, and SPSS 25.0 software was used for statistical analysis. Data were expressed as mean ± s. To reduce experimental errors, the above process was repeated 3 times. A one-way analysis of variance (ANOVA) was used to compare between the groups, and the mean comparison between the two groups was tested using a t-test. *p* < 0.05 indicated a statistically significant difference.

## 3. Results

### 3.1. Rate of growth and alterations in the morphology

**Figure 1.**
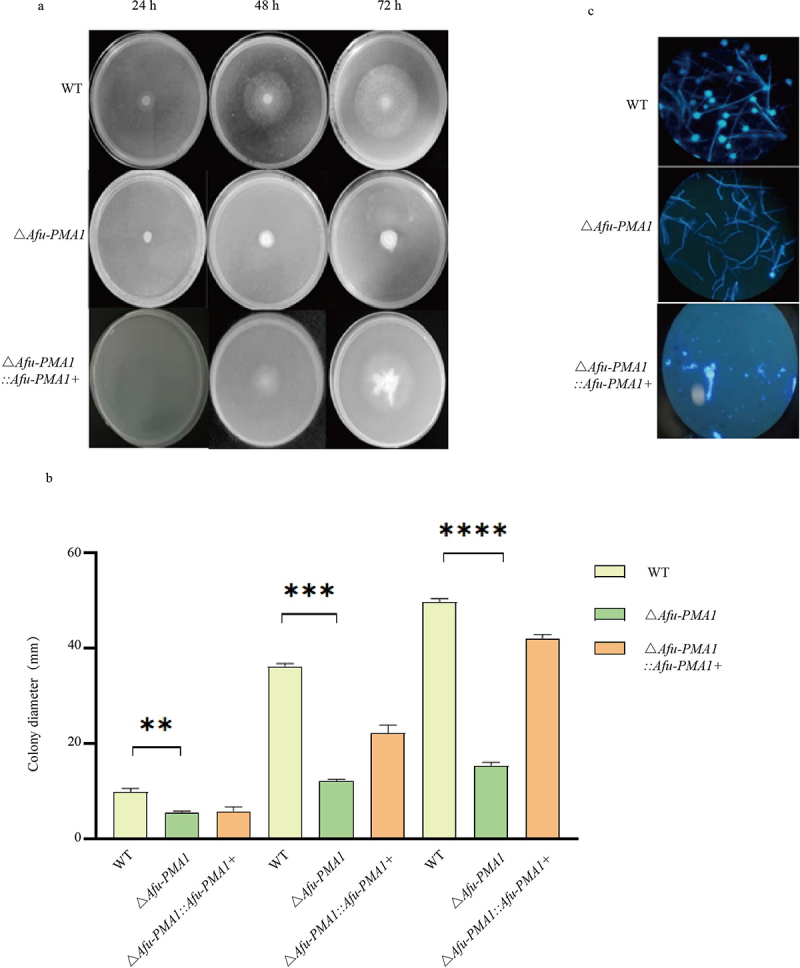
Growth phenotype. (a) Diameter of the strains. (b) Comparison of colony diameters (**: *p* = 0.0093, ***: *p* = 0.001, ****: *p* < 0.0001). (c) Fluorescent dye examination for conidia and hyphae.

The results showed that the Δ*Afu-PMA1* colonies were significantly smaller compared to the WT colonies (Figure 1a). Statistical analysis of the colony diameter measurements at different time points revealed significant differences in growth between Δ*Afu-PMA1* and WT (*p* < 0.05), as shown in Figure 1b. This indicated that the difference in the growth rate between Δ*Afu-PMA1* and WT was primarily caused by the absence of the *PMA* gene.

To observe the morphology of *A. fumigatus*, fungal fluorescent staining was performed, and the samples were examined using a fluorescence microscope at a 40× magnification level (Figure 1c). The results showed that compared to WT, Δ*Afu-PMA1* exhibited thinner and shorter hyphae, a reduced number of conidiophores at the tips of hyphae, and no prominent branching. The results showed that the deletion of the *PMA* gene affected the morphology of *A. fumigatus*.

### 3.2. Determination of drug sensitivity

**Figure 2.**
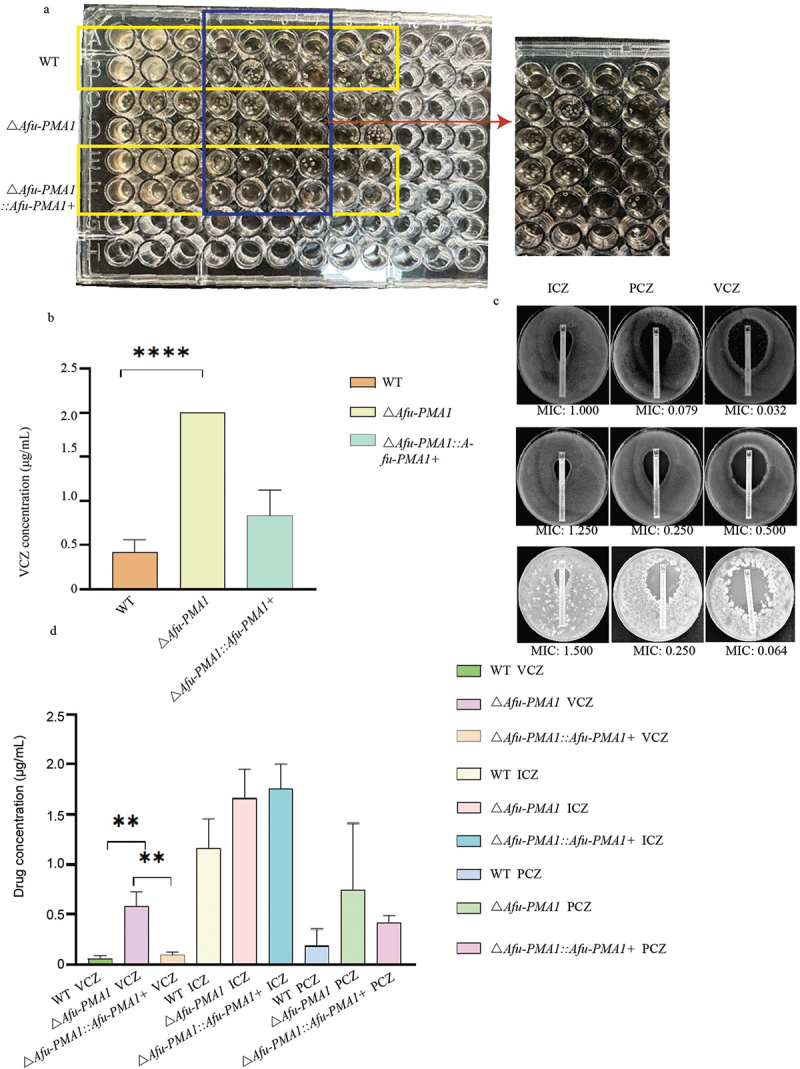
Antifungal susceptibility test. (a) The antifungal susceptibility of voriconazole (VCZ) by microdilution. (b) The MICs of VCZ, ****: *p* < 0.0001. (c) The results of E-test for test strains. (d) The MICs of E-test, **: *p* = 0.0035.

The microdilution assay of Δ*Afu-PMA1* showed significant resistance to voriconazole, in contrast to both WT and Δ*Afu-PMA1::Afu-PMA1+* (Figure 2a). The MIC for WT was 0.25 μg/mL, while the MIC for Δ*Afu-PMA1* was 2.00 μg/mL, representing an 8-fold increase compared to WT (*p* < 0.0001) (Figure 2b) (Table 2). However, no significant difference in susceptibility was observed between WT and Δ*Afu-PMA1* for itraconazole and posaconazole.

**Table 2.** Effects of *DAfu-PMA1* on *in vitro* antifungal susceptibility profiles.

Strain	MIC (mg/mL)						
	Via broth microdilution				Via E-test		
	ICZ	VCZ	PCZ		ICZ	VCZ	PCZ
WT	1	0.25	1		1	0.032	0.38
*DAfu-PMA1*	1	2	1		2	0.75	0.5
*DAfu-PMA1::Afu-PMA1+*	1	1	1		1.75	0.064	0.38

Consistent with the results of the microdilution assay, the E-test results for WT, Δ*Afu-PMA1::Afu-PMA1+*, and Δ*Afu-PMA1* also showed similar trends (Figure 2c). The MIC of voriconazole for WT was 0.052 μg/mL, while the MIC for Δ*Afu-PMA1* was 0.583 μg/mL, representing an 11.21-fold increase in drug concentration compared to WT (*p* = 0.0035) (Figure 2d). Both of these susceptibility tests indicated that Δ*Afu-PMA1* exhibited resistance to voriconazole in comparison to WT (Table 2).

### 3.3. Determination of oxidative stress

**Figure 3.**
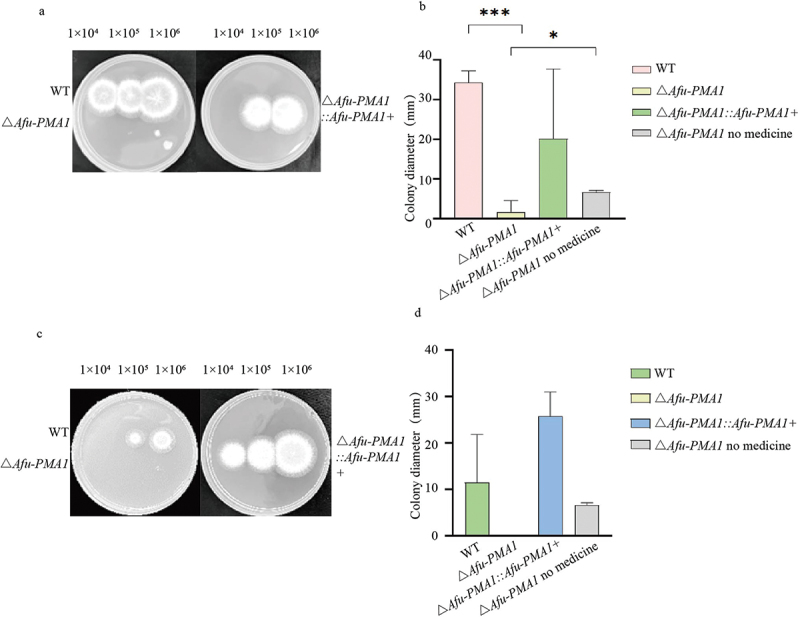
Oxidative stress. (a) The growth phenotype of WT, Δ*Afu-PMA1*, and Δ*Afu-PMA1::Afu-PMA1+* strains on the medium supplemented with hydrogen peroxide. (b) Results of hydrogen peroxide experiments (***: *p* = 0.0002, *: *p* = 0.0448). (c) The growth phenotype of WT, Δ*Afu-PMA1*, and Δ*Afu-PMA1::Afu-PMA1+* strains on the media supplemented with menadione. (d) Results of menadione experiment.

After 48 h of incubation, the diameter of the colonies was measured (Figure 3a). The results showed a significant difference (*p* = 0.0002) in growth between Δ*Afu-PMA1* and WT when exposed to H_2_O_2_ (Figure 3b), with a significant poor growth observed in Δ*Afu-PMA1* compared to WT. When the concentration of menadione was 25 mmol/L, the colony diameter of Δ*Afu-PMA1* was significantly smaller compared to WT (Figure 3c). Menadione exhibited a more pronounced inhibitory effect on Δ*Afu-PMA1*, but the difference was not statistically significant (*p* = 0.125) (Figure 3d). Both sets of experiments indicated that Δ*Afu-PMA1* reduced the resistance to the oxidising agents H_2_O_2_ and menadione. When Δ*Afu-PMA1* grown in an H_2_O_2_-added medium was compared with Δ*Afu-PMA1* grown in a medium without H_2_O_2_, a significant difference was observed (*p* = 0.0448).

### 3.4. Osmotic pressure test

**Figure 4.**
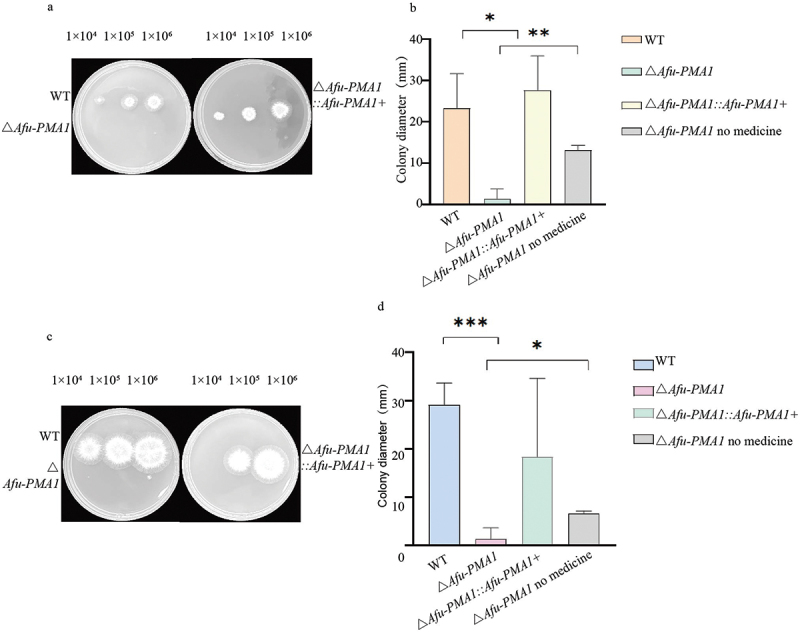
Osmotic pressure test. (a) Growth phenotype of WT, Δ*Afu-PMA1*, and Δ*Afu-PMA1::Afu-PMA1+* strains on Nacl-supplemented medium. (b) Results of Nacl experments (*: *p* = 0.012, **: *p* = 0.0016). (c) Growth phenotype of WT, Δ*Afu-PMA1*, and Δ*Afu-PMA1::Afu-PMA1+* strains on the medium supplemented with sorbitol. (d) Results of sorbitol experiment (***: *p* = 0.0007, *: *p* = 0.0189).

After 48 h of incubation, the diameter of the colonies was measured (Figure 4a). After adding NaCl, the colony diameter of the Δ*Afu-PMA1* strain was noticeably smaller than that of the WT (Figure 4b) (*p* < 0.05). Upon the addition of D-sorbitol, the diameter of the Δ*Afu-PMA1* strain significantly decreased compared to WT (Figure 4c), indicating a higher sensitivity to D-sorbitol. This observed difference was statistically significant (Figure 4d) (*p* = 0.0007), suggesting that the deletion of the *PMA* gene reduced the strain’s resilience to osmotic pressure. In comparison to Δ*Afu-PMA1* grown in media without NaCl and D-sorbitol, significant differences were observed in Δ*Afu-PMA1* grown in media containing NaCl and D-sorbitol.

### 3.5. Determination of ergosterol content

**Figure 5.**
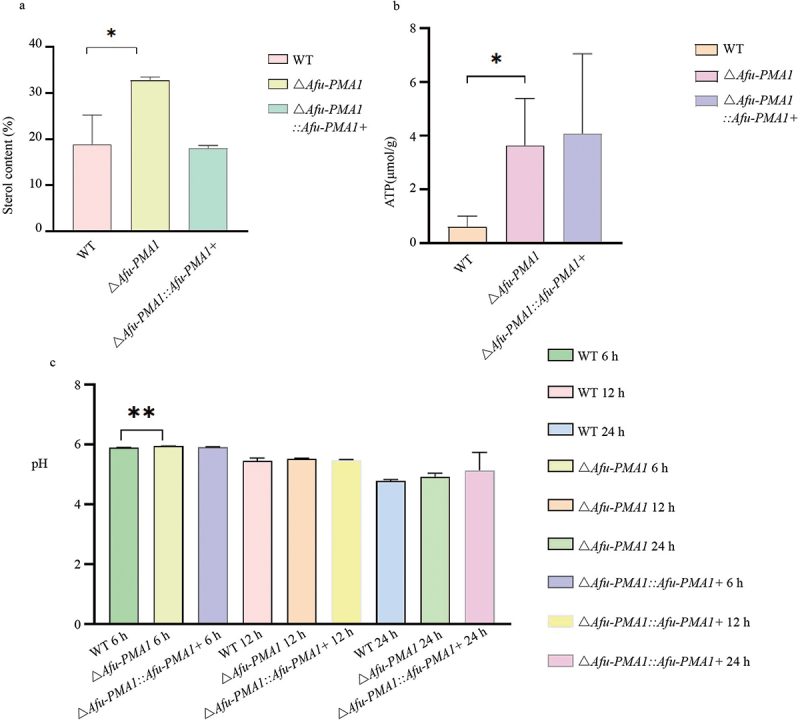
Sterol, ATP, environmental pH determination. (a) WT, Δ*Afu-PMA1*, and Δ*Afu-PMA1::Afu-PMA1+* strains were used to determine the content of sterol (*p* = 0.0198). (b) Determination of ATP in WT, Δ*Afu-PMA1*, and Δ*Afu-PMA1::Afu-PMA1+* strains (*p* = 0.0431). (c) The pH value of growth environment of WT, Δ*Afu-PMA1*, and Δ*Afu-PMA1::Afu-PMA1+* strains in different time (*p* = 0.008).

The results of ergosterol extraction and analysis for WT, Δ*Afu-PMA1*, and Δ*Afu-PMA1::Afu-PMA1+* are shown in Figure 5a. A significant difference in ergosterol content was observed between WT and Δ*Afu-PMA1* (*p* = 0.0198); the content of ergosterol was higher in Δ*Afu-PMA1* compared to WT. The *PMA* gene might associate with the synthesis of fungal ergosterol, and its knockout led to an increase in *A. fumigatus* ergosterol synthesis.

### 3.6. Measurement of intracellular ATP levels

The analysis of ATP extraction data for WT, Δ*Afu-PMA1*, and Δ*Afu-PMA1::Afu-PMA1+* strains is shown in Figure 5b. It can be observed that there was a significant difference in ATP levels between WT and Δ*Afu-PMA1* (*p* = 0.0431). This suggests that *PMA* plays an important role in ATP synthesis or utilisation, as the detected ATP levels in the fungus significantly increased when the gene was knocked out.

### 3.7. Effect of environmental pH

The results of the data analysis showed a significant difference (Figure 5c) (*p* = 0.008) in pH values between WT and Δ*Afu-PMA1::Afu-PMA1+* at a growth time of 6 h. The pH value of the environment for the Δ*Afu-PMA1* strain was higher than that for WT, indicating a correlation between the *PMA* gene and the regulation of environmental pH in *A. fumigatus.*

### 3.8. RNA sequencing

**Figure 6.**
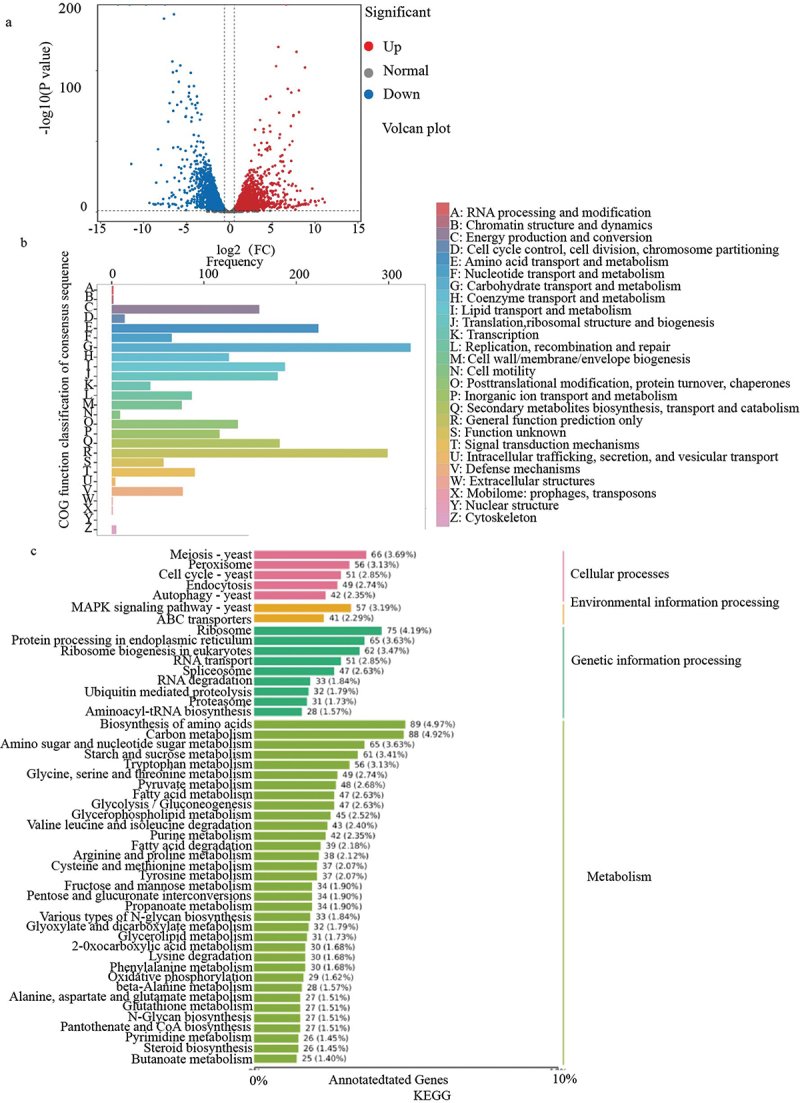
RNA sequencing results. (a) Each point in the figure represents a gene, and the horizontal coordinate represents the logarithmic value of the expression difference multiple of a certain gene in two samples. The ordinate represents the negative value of the statistical significance of the change in gene expression. (b) The horizontal coordinate is the classification content of COG, and the vertical coordinate is the number of genes. (c) The left vertical coordinate is the name of the KEGG metabolic pathway, the right vertical coordinate represents the first-class classification name corresponding to the annotated pathway, and the horizontal coordinate is the number of genes annotated to the pathway and their proportion to the total number of genes annotated.

RNA sequencing results revealed that there were 5,595 genes altered in Δ*Afu-PMA1* when compared to WT, with 2,754 genes being up-regulated and 2,841 genes being down-regulated (Figure 6a). The differential expression gene COG classification statistics show that the genes related to carbohydrate transport and metabolism, amino acid transport and metabolism, energy production and conversion, lipid transport and metabolism, and translation, ribosomal structure and biogenesis were reduced in Δ*Afu-PMA1* compared to WT (Figure 6b). In the KEGG map of *ΔAfu-PMA1* and WT differentially expressed genes, *ΔAfu-PMA1* exhibits lower gene expression levels compared to WT in areas such as meiosis, MAPK signalling pathway – yeast, ribosome, protein processing in the endoplasmic reticulum, biosynthesis of amino acids and carbon metabolism (Figure 6c). The RNA sequencing results suggest that the genes reduced in Δ*Afu-PMA1* were mainly concentrated in the processes of nutrient metabolism and transport, ATP utilisation, and growth and development, which are relatively energy-consuming.

## 4. Discussion

The *PMA* gene is responsible for encoding the plasma membrane H (+) ATPase, a protein that is typically anchored on the fungal cell membrane to establish an electrochemical gradient, thus functioning as an energy source (Burghoorn et al. 2002). H (+) ATPase belongs to the P-type ATPase family, which is a highly conserved and extensive family characterised by a uniform overall protein structure and the formation of a phosphorylated intermediate during the transportation process (Pedersen et al. 2018). As expected, after the deletion of the *PMA* gene, the amount of ATP increased significantly, which must lead to a series of physiological process to change. The results of this study indicate that the *PMA* gene is essential for the growth and voriconazole sensitivity of *A. fumigatus.*

Ergosterol is the main sterol component in fungal cell membranes and plays an important role in maintaining the integrity of cell membranes (Choy et al. 2023). Voriconazole’s antifungal mechanism involves inhibiting 14-alpha-demethylase and further suppressing ergosterol to disrupt cell membrane integrity, thereby achieving antifungal effects (Kalamkar et al. 2013; Shishodia et al. 2019). The determination of ergosterol content showed that *PMA* gene knockout led to the increase of ergosterol content which further promoted the resistance to voriconazole. After *PMA* deletion, Δ*Afu-PMA1* showed obvious growth inhibition compared to WT, as well as defect growth under oxidative and osmotic stress. This was likely a result of the compromised function of the plasma membrane H (+)-ATPase in utilising ATP, which, in turn, led to a reduction in the pumping of H^+^ ions and a decreased capacity to acidify the environment (Mariscal et al. 2022). H (+)-ATPase pumping H^+^ is important for the growth of fungi, on the one hand forming an electrochemical gradient, which is critical for the transport capacity of substances (Burghoorn et al. 2002; Clausen et al. 2017; Kjellerup et al. 2017). On the other hand, pumping H^+^ can regulate the pH of the environment, and the stronger the regulation ability, the faster the growth of fungi in the same environment (Burghoorn et al. 2002; Zhang et al. 2023). In our experiment, we observed an increased environmental sensitivity in Δ*Afu-PMA1*, which aligns with the conclusions drawn by other researchers that the absence of *PMA* leads to a reduced ability to cope with oxidative stress and osmotic pressure changes (Jia et al. 2018; Velle et al. 2023).

From the results of RNA sequencing, it is evident that Δ*Afu-PMA1* exhibits reduced expression in the areas related to the metabolism and transport of sugars, lipids, and amino acids, as well as energy production and conversion. This aligns with the experimental results showing an increase in ergosterol and ATP levels in Δ*Afu-PMA1*. In the KEGG pathway, there is a decrease in the expression of meiosis, MAPK signalling pathway – yeast, ribosome, protein processing in the endoplasmic reticulum, biosynthesis of amino acids, and carbon metabolism. Ribosome, MAPK, and biosynthesis of amino acids are closely associated with fungal growth and environmental adaptation, which is consistent with the results we observed in this experiment showing a slowdown in Δ*Afu-PMA1* growth and a decrease in its ability to adapt to the environment (Kurata et al. 2010; Greber and Ban 2016; Gurgel et al. 2019; Abdulghani et al. 2022).

H (+) ATPase is located at the last stage of the entire energy chain, where it breaks down ATP produced by other organelles to support various activities that require energy consumption (Zhang et al. 2023). In this study, after the deletion of the *PMA* gene, the ability to utilise ATP was weakened, resulting in significantly higher ATP content in the Δ*Afu-PMA1* than in WT. Obstacles in ATP use can cause fungi to show slower growth and reduced environmental adaptability, which is also consistent with what other researchers have found (Kurata et al. 2010; Greber and Ban 2016; Tiwari et al. 2016; Gurgel et al. 2019; Abdulghani et al. 2022).

Our research provided evidence that the *PMA* gene in *A. fumigatus* influences the strain’s growth and susceptibility to voriconazole. This impact stems from its role in regulating the ability of fungal cells to respond to environmental stress, meet nutritional requirements, break down ATP, and synthesise sterols. These findings have contributed to a deeper understanding of the growth and drug resistance mechanisms of *A. fumigatus*. And also provide potential implications for targeting the *PMA* as a strategy for developing antifungal drugs.

## 5. Conclusions

Following the knockout of the *PMA* gene, notable alterations in the growth and morphology of *A. fumigatus* were observed. The growth rate of Δ*Afu-PMA1* exhibited a significant deceleration, accompanied by morphological changes in conidia. Moreover, under conditions of oxidative stress and osmotic pressure, Δ*Afu-PMA1* displayed a marked decrease in the growth rate, indicative of its heightened susceptibility to environmental stressors. Additionally, Δ*Afu-PMA1* exhibited impaired acidification ability within its environment. Elevated levels of ergosterol and ATP content were noted in Δ*Afu-PMA1*, concomitant with a heightened resistance to voriconazole. These observations underscore the pivotal role of the *PMA* gene in regulating drug sensitivity and fungal membrane composition. Furthermore, the downregulation of genes associated with nutrient transport, metabolism, ribosome function, the MAPK pathway, and endoplasmic reticulum function in Δ*Afu-PMA1* highlights the broad regulatory influence of *PMA* on essential cellular processes. These findings underscore the significance of the *PMA* gene in orchestrating adaptive responses to environmental stimuli and modulating drug sensitivity in *A. fumigatus*. Notably, our results suggest the potential of targeting *PMA* as a promising strategy for antifungal drug development.
