# Correction

**DOI:** 10.1080/21501203.2025.2461914

**Published:** 2025-02-06

**Article title**: Exploring the role and mechanisms of the PMA gene in Aspergillus fumigatus

**Authors**: C. Tan., S. Jiang., H. Zhai., Q. Hu., C. Liu., Y. Sun & L. Gao

**Journal**: *Mycology*

**DOI**: https://doi.org/10.1080/21501203.2024.2354273

This article was previously published but contained an error in the references.

This has now been added and republished in the original article.

Ma DM, Ji YJ, Yang F, Liu W, Wan Z, Li RY. 2012. The effect of extra copies of sho1 or pbs2 gene on the adaptive ability of Aspergillus fumigatus to several stresses. Chinese Journal of Mycology. 7:193-198 (in Chinese).

Ma Y, Qiao JJ, Liu W, Li RY. 2009. Effect of sho1 gene of Aspergillus fumigatus on adaptation to osmotic pressure and on sensitivity of antifungal drugs. Journal of Peking University (Health Sciences). 41:162-167. doi:10.3969. (in Chinese).

Liu LY, Liao WZ, Zhang H, Gao LJ, Li LY, Sun Y. 2022. Potential contribution of FADB to drug resistance in Aspergillus fumigatus. Mycosystema. 41(04):561-569. doi:10.13346/j.mycosystema.210326. (in Chinese).

